# Targeting proliferative retinopathy: Arginase 1 limits vitreoretinal neovascularization and promotes angiogenic repair

**DOI:** 10.1038/s41419-022-05196-8

**Published:** 2022-08-29

**Authors:** Abdelrahman Y. Fouda, Zhimin Xu, Jutamas Suwanpradid, Modesto Rojas, Esraa Shosha, Tahira Lemtalsi, Chintan Patel, Ji Xing, Syed A. Zaidi, Wenbo Zhi, Brain K. Stansfield, Paul Ning-Man Cheng, S. Priya Narayanan, R. William Caldwell, Ruth B. Caldwell

**Affiliations:** 1 University of Arkansas for Medical Sciences, Little Rock, AR USA; 2 Department of Clinical Pharmacy, Faculty of Pharmacy, Cairo University, Cairo, Egypt; 3 Vascular Biology Center, Augusta University, Augusta, GA USA; 4 Culver Vision Discovery Institute, Augusta University, Augusta, GA USA; 5 Department of Pharmacology and Toxicology, Augusta University, Augusta, GA USA; 6 Department of Cellular Biology & Anatomy, Augusta University, Augusta, GA USA; 7 Center for Biotechnology and Genomic Medicine, Augusta University, Augusta, GA USA; 8 Department of Pediatrics, Augusta University, Augusta, GA USA; 9 Bio-cancer Treatment International, 511-513, Bioinformatics Building, Hong Kong Science Park, Tai Po, Hong Kong SAR China; 10 Department of Clinical and Administrative Pharmacy, University of Georgia, Augusta, GA USA; 11 Charlie Norwood VA Medical Center, Augusta, GA USA

**Keywords:** Translational research, Preclinical research, Neuro-vascular interactions

## Abstract

Current therapies for treatment of proliferative retinopathy focus on retinal neovascularization (RNV) during advanced disease and can trigger adverse side-effects. Here, we have tested a new strategy for limiting neurovascular injury and promoting repair during early-stage disease. We have recently shown that treatment with a stable, pegylated drug form of the ureohydrolase enzyme arginase 1 (A1) provides neuroprotection in acute models of ischemia/reperfusion injury, optic nerve crush, and ischemic stroke. Now, we have determined the effects of this treatment on RNV, vascular repair, and retinal function in the mouse oxygen-induced retinopathy (OIR) model of retinopathy of prematurity (ROP). Our studies in the OIR model show that treatment with pegylated A1 (PEG-A1), inhibits pathological RNV, promotes angiogenic repair, and improves retinal function by a mechanism involving decreased expression of TNF, iNOS, and VEGF and increased expression of FGF2 and A1. We further show that A1 is expressed in myeloid cells and areas of RNV in retinal sections from mice with OIR and human diabetic retinopathy (DR) patients and in blood samples from ROP patients. Moreover, studies using knockout mice with hemizygous deletion of A1 show worsened RNV and retinal injury, supporting the protective role of A1 in limiting the OIR-induced pathology. Collectively, A1 is critically involved in reparative angiogenesis and neuroprotection in OIR. Pegylated A1 may offer a novel therapy for limiting retinal injury and promoting repair during proliferative retinopathy.

## Introduction

Ischemic retinopathies, such as diabetic retinopathy (DR) and retinopathy of prematurity (ROP), are major causes of blindness in adults and neonates, respectively. Both conditions are characterized by vaso-obliteration and lack of adequate vascular repair. This leads to relative hypoxia, which induces upregulation of pro-inflammatory and pro-angiogenic growth factors and promotes pathological retinal neovascularization (RNV). Laser photocoagulation is usually effective for treatment of advanced retinopathy but can impair vision and in some patients, the pathology continues to progress. Intra-ocular injections with anti-vascular endothelial growth factor (VEGF) agents show promise in both DR [1] and severe late-stage ROP [2, 3]. However, neither treatment promotes tissue repair and there is a potential risk of adverse effects and disease recurrence with anti-VEGF therapy [4, 5]. Thus, there is a great need for new therapies to limit the neurovascular injury and promote repair.

Our previous studies have shown that expression of the ureohydrolase enzyme arginase is critically involved in retinal injury and repair in models of retinopathy [6–11]. Arginase has two isoforms, arginase 1 (A1), which is cytosolic, and arginase 2 (A2), which is mitochondrial [12]. The two isoforms have very similar mechanisms of action. Both metabolize arginine to produce urea and ornithine. However, they differ substantially in terms of their tissue distribution and involvement in disease and injury. Arginase 1 is highly expressed in the liver where it plays a critical role in the urea cycle. Mutations in A1 can result in hyperammonemia and A1 global knockout mice die soon after birth. A2 is highly expressed in the kidney but is also expressed in many other tissues. In contrast with A1 deficiency, mice globally deficient in A2 show no noticeable phenotype. Studies have shown that A1 plays a key role in the repair phase of wound healing, whereas A2 has been implicated in chronic inflammatory disease conditions [12].

Both isoforms have also been implicated in retinal injury and repair. Our studies in mouse models of oxygen-induced retinopathy (OIR), retinal ischemia/reperfusion injury, and optic nerve crush have demonstrated the involvement of the A2 isoform in neurovascular injury. We showed that deletion of the A2 gene significantly reduced both neuronal and vascular injury during OIR, while enhancing vascular repair and limiting pathological RNV [6–8]. Studies using the ischemia/reperfusion and optic nerve crush models showed retinal protection with A2 gene deletion [9, 10]. In contrast, A1 deletion in A2 deficient mice with OIR or A1 deletion in the ischemia/reperfusion mouse model amplified the signs of injury [8, 11]. Consistently, studies in acute models of ischemia/reperfusion, optic nerve crush, or ischemic stroke showed that treatment with a stable (pegylated) form of A1 limited neuronal injury [11, 13]. Here, we have characterized the specific effects of this A1 treatment on RNV, vascular repair, and neuronal function in the OIR model and examined the underlying mechanisms.

## Materials and methods

Owing to word limit, only main experiments are described here and detailed methods for the rest of experiments together with full (uncropped) Western blots are included in the supplementary file.

### OIR mouse model

OIR was induced in newborn mice according to the protocol of Smith, et al. with some adjustment [14, 15]. On P7, pups of both sexes were placed along with their dams in a hyperoxia (75% oxygen) chamber for up to 5 days, after which they were transferred back to room air (RA, 21% oxygen) on P12. The OIR model is characterized by vaso-obliteration of the central retinal vessels (P7-P12) followed by vascular regrowth (P12-P17) and pathological retinal neovascularization (RNV, P14-P17) [15]. Controls were maintained in RA. Mice were sacrificed during the periods of vaso-obliteration, regrowth, and RNV (Fig. S1).

Mice were compared to their littermate controls and therefore no specific randomization scheme was used. Data were pooled together to minimize variability between litters due to differences in litter size/weight [16]. Both sexes were combined given the lack of differences between sexes. Experiments were performed in accordance with the ARVO Statement for the Use of Animals in Ophthalmic and Vision Research and were approved by the institutional animal care and use committee (Animal Welfare Assurance no. A3307–01).

### PEG-A1 treatment in OIR

A pharmaceutical grade of PEG-A1 was a kind gift from Bio-Cancer Treatment International Limited (BCT, Hong Kong). PEG-A1 is a recombinant human arginase (rhArg) covalently attached to methoxy polyethylene glycol (mPEG-SPA; MW 5000) to increase its stability and half-life in vivo, which has been reported to be 3 days vs. a few minutes for the native enzyme [17]. PEG-A1 was prepared from a 3.4 mg/mL stock by dilution in PBS (1:250 ratio) to achieve final concentration of 13.6 ng/μL. PBS was used as vehicle control. Intravitreal injections were performed on anesthetized pups using a 36-gauge NanoFil needle mounted to a 10-μL Hamilton syringe (World Precision Instruments). Two treatment strategies were employed. To examine vaso-obliteration, wildtype (WT, C57BL6J) pups received single intravitreal injection of PEG-A1 (6.8 ng in 0.5 μL—based on a preliminary dose/response study) at P7 then subjected to hyperoxia (75% oxygen) for 2 days and sacrificed at P9. The P9 time point was selected based on the fact that vaso-obliteration occurs within the first 48 h of hyperoxia treatment [15, 18, 19]. Another cohort of WT pups was placed in hyperoxia (75% oxygen) on P7, switched to RA on P12, immediately given single intravitreal PEG-A1 injections (6.8 ng in 0.5 μL), and sacrificed on P17 (Fig. S1A).

### A1 deletion in OIR

To assess the specific role of A1 expression in OIR, we used A1 knock out mice and WT littermates (Fig. S1B) [20–22]. As deletion of both copies of A1 is lethal due to hyperammonemia, we used heterozygous mice lacking 1 copy of A1 (A1^+/−^ or A1 KO), which is sufficient to dampen its activity [20–22]. These mice develop normally. A 70% oxygen concentration was used for experiments involving A1 KO mice based on preliminary experiments showing intolerance of the A1 KO mice to hyperoxia treatment.

Myeloid and endothelial cell-specific A1 KO mice were used in this study under LysM cre and Cdh5 cre respectively as described and characterized before [11].

### Statistical analysis

Sample size was determined based on our previous experience with the OIR model. Outliers were determined and excluded based on GraphPad Prism outliers calculator. Data were analyzed by investigators blinded to the group identity. Statistical analysis was conducted using GraphPad Prism 9 software. Differences between two groups were determined by student’s *t*-test. Differences between multiple groups were analyzed by ANOVA with Tukey’s post hoc test. *P*-values < 0.05 were considered statistically significant. Graphs were prepared using GraphPad Prism 9 software and data were presented as mean ± standard deviation (SD).

### Reporting summary

Further information on research design is available in the Nature Research Reporting Summary linked to this article.

## Results

### A1 treatment decreases pathological neovascularization while promoting reparative angiogenesis

Based on our previous findings that A1 deletion worsens retinal pathology in ischemia/reperfusion injury and that treatment with A1 limits neuronal injury in models of ischemia/reperfusion, optic nerve crush, and acute stroke [8, 11, 13], we hypothesized that the A1 therapy could be effective in limiting the OIR-induced retinal injury. To test this, we used a PEGylated form of A1 (PEG-A1). We established the pharmacokinetic profile of PEG-A1 in the vitreous of adult mice by measuring arginase activity after single intravitreal injection of PEG-A1 (6.8 ng in 1 μL). Adult mice were used to get adequate vitreous yield for analysis. Immediately after intravitreal administration, vitreous arginase activity was increased by about sixfold as compared to vehicle treatment. Arginase activity remained significantly elevated (three-fold) at day 3 post-injection and returned to baseline at day 6 (Fig. S2A).

**Fig. 1:**
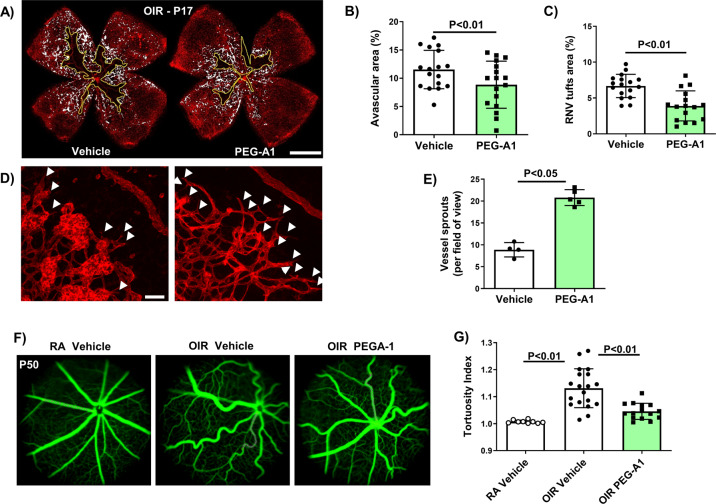
PEG-A1 promotes vessel sprouting and reduces the AVA in OIR retinas at P17. **A**–**C** OIR mice were injected intravitreally with either PEG-A1 or vehicle on P12 and sacrificed at p17. Quantitation of lectin-labeled retinal flatmounts showed significant decreases in AVA and RNV tuft formation with PEG-A1 treatment. Scale bar = 100 µm. **D**, **E** Higher magnification images and quantification showed a significant increase in vessel sprouts (white-arrow heads) entering the AVA with PEG-A1 treatment. Scale bar = 20 µm. **F**, **G** Fluorescein angiography at P50 showed normalization of OIR-induced vessel tortuosity with PEG-A1 treatment.

We determined the effects of the PEG-A1 treatment on OIR-induced pathological RNV along with physiological vascular repair during the relative hypoxia phase of OIR. WT pups were subjected to 5 days of hyperoxia from P7 to P12, switched to RA, and treated with intravitreal PEG-A1 (single injection, 6.8 ng in 0.5 μL) on P12. Retinas were collected on P17 and flatmounts labeled with isolectin B4 were prepared. Morphometric analysis showed a significant reduction in the area of pathological RNV tufts in the PEG-A1-treated retinas. Moreover, the avascular area (AVA) was also reduced with PEG-A1 treatment, indicating an increase in reparative angiogenesis leading to revascularization (Fig. 1A–C). In accordance with this, the PEG-A1 treatment resulted in a twofold increase in endothelial tip cells (vessel sprouts) per field of view (Fig. 1D, E).

Analysis of vascular tortuosity, a clinical sign of severe ROP [23], using fluorescein angiography showed prominent and persistent vascular tortuosity after the OIR exposure that was clearly evident at P50 (Fig. 1F, G).The reduction in pathological RNV and improved vascular repair seen with PEG-A1 treatment were associated with a significant reduction in vascular tortuosity.

We next evaluated the effects of PEG-A1 on hyperoxia-induced vaso-obliteration. WT pups were injected intravitreally with PEG-A1 (single injection, 6.8 ng in 0.5 μL) at P7 and subjected to hyperoxia for 2 days. Morphometric analysis of P9 retina flatmounts labeled with isolectin B4 showed no change in the area of vaso-obliteration with PEG-A1 treatment as compared to vehicle-treated retinas (Fig. S2B, C). However, the PEG-A1-treated retinas showed a significant increase in A1 protein levels (Fig. S2D, E) and Iba1/lectin-positive macrophage/microglial cells at the borders of the central avascular zone (Fig. S2F, G). These results indicate that the A1 treatment does not alter the hyperoxia-induced injury, but that A1 expression may have a role in the subsequent repair process.

### A1 treatment protects against OIR-induced retinal injury and dysfunction

**Fig. 2:**
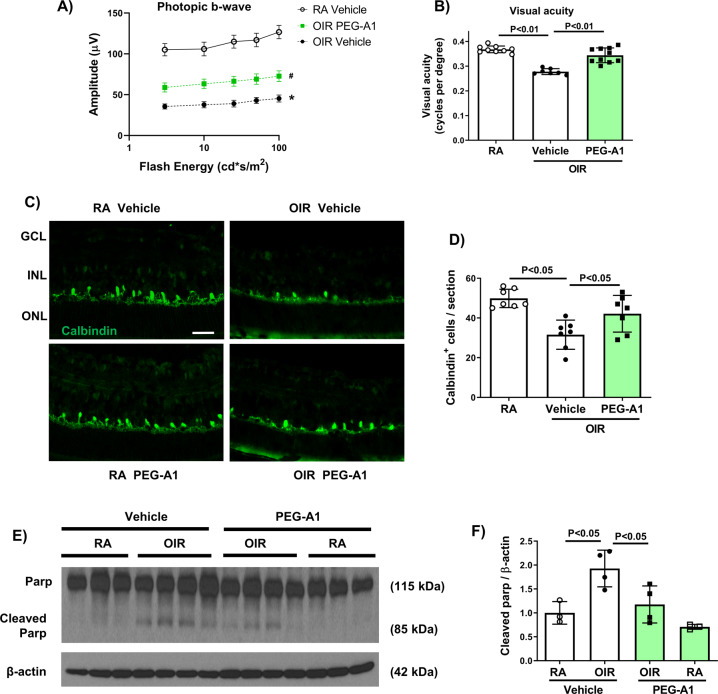
PEG-A1 treatment reduces OIR-induced retinal dysfunction, limits loss of horizontal cells, and reduces PARP cleavage. **A** OIR reduced the retinal photopic B-wave ERG response at P50, which was partially rescued by PEG-A1 treatment. **B** OIR reduced visual acuity at P50 as measured by OptoMotry and this was rescued by PEG-A1 treatment. **C**, **D** Immunofluorescence labeling of P17 retinal sections with the horizontal cell marker Calbindin at P17 showed a decrease in numbers of horizontal cells in Vehicle-OIR retinas as compared to RA. PEG-A1 treatment reduced this loss. Scale bar = 50 µm. **E**, **F** Western blotting and quantification of P17 retinal lysates showed increased PARP cleavage in the OIR retinas, which was significantly reduced with PEG-A1 treatment.

In order to determine how PEG-A1 treatment affected retina function, we subjected WT mice to OIR, treated them with PEG-A1 on P12 and performed ERG recordings at P50. OIR mice showed reduced photopic b-wave responses, which were improved with PEG-A1 treatment (Fig. 2A). Mice treated with PEG-A1 also exhibited improved visual acuity as compared to vehicle treatment (Fig. 2B). The improved retinal function after PEG-A1 treatment was accompanied by reductions in neuronal injury as indicated by improved survival of calbindin-positive horizontal cells (Fig. 2C, D) along with a significant decrease in levels of the pro-apoptotic marker, cleaved PARP (Fig. 2E, F).

### A1 treatment ameliorates the OIR-induced inflammatory response and reduces VEGF while increasing FGF2

**Fig. 3:**
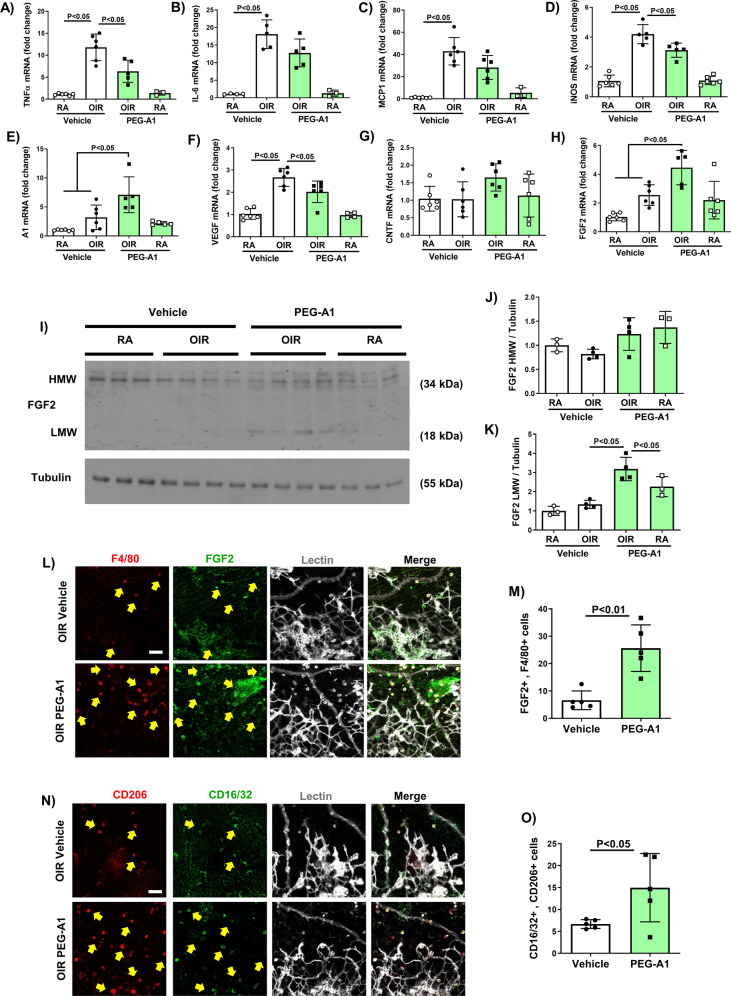
PEG-A1 treatment ameliorates the OIR-induced inflammatory response and reduces VEGF while increasing FGF2. **A**–**H** Analysis of mRNA levels showed upregulation of TNF, IL-6, MCP1, and VEGF in OIR retinas at P13. PEG-A1 treatment significantly reduced TNF and VEGF and showed a trend towards a reduction in IL-6 and MCP1. PEG-A1 treatment also showed a trend towards increasing the neurotrophic factor CNTF at P13. Analysis at P17 showed upregulation of iNOS mRNA after OIR, which was significantly reduced with PEG-A1 treatment. Furthermore, PEG-A1 treatment increased the mRNA levels of A1 and FGF2 at P17. **I**–**K** Western blotting of retina lysates at P17 showed upregulation of low molecular weight (LMW) FGF2 (18 kDa band) with PEG-A1 treatment as compared to vehicle with a similar increase in the high molecular weight (HMW) FGF2 but the later did not reach statistical significance. **L**, **M** Immunolabeling of retina flatmounts at P17 showed co-localization of FGF2 with F4/80 (microglia/macrophage marker) and lectin (blood vessel marker) (arrows). Image quantification showed upregulation of FGF2/F4/80 double-positive cells with PEG-A1 treatment. Scale bar = 20 µm. **N**, **O** Immunolabeling of retina flatmounts at P17 showed co-localization of the M2 macrophage marker CD206 with the M1 marker CD16/32. Image quantification showed upregulation of CD16/32 / CD206 double-positive cells with PEG-A1 treatment. Scale bar = 20 µm.

To examine the effect of PEG-A1 on OIR-induced inflammation markers and angiogenic factors, we performed qPCR on retina samples collected from mice treated with PEG-A1 or vehicle on P12 and sacrificed at P13 or P17. OIR retinas showed upregulation of the inflammatory markers, inducible nitric oxide synthase (iNOS), tumor necrosis factor (TNF), interleukin (IL-) 6, and monocyte chemoattractant protein 1 (MCP1). PEG-A1 treatment reduced these increases. However, variability was relatively high in the OIR retinas, and this protective effect was statistically significant only for iNOS and TNF (Fig. 3A–D). Furthermore, PEG-A1 treated OIR retinas showed downregulation of mRNA for vascular endothelial growth factor (VEGF) and upregulation of that for A1, fibroblast growth factor 2 (FGF2), and ciliary neurotrophic factor (CNTF, albeit not statistically significant). Both FGF2 and CNTF have been found to facilitate physiological revascularization (Fig. 3E–H) [24, 25]. Western blotting analysis confirmed the increase in FGF2 and decreases in iNOS and VEGF with PEG-A1 treatment in OIR retinas (Fig. 3I–K, Fig. S2H–K). Flatmount immunolabeling showed co-localization of FGF2 with blood vessels and microglia/macrophages in and around the pathological RNV tufts as well as in the zone of tip cell formation. PEG-A1 treatment increased the number of FGF2 and F4/80 double-positive microglia/macrophages in both regions (Fig. 3L, M). Further analysis using markers for M1, and M2-like macrophage/microglia showed an increase in double-positive cells in the areas of tip cell formation following PEG-A1 treatment (Fig. 3N, O).

### Global A1 deletion increases OIR-induced pathological neovascularization, reduces vascular repair, and worsens vascular tortuosity

Taken together with our previous finding that the protective effects of the A2 deletion were abrogated in double KO mice that lacked one copy of the A1 isoform as well as both copies of A2 [8], the above noted protective actions of PEG-A1 suggest that A1 plays a critical role in limiting OIR-induced neurovascular injury. We tested this by studies using A1^+/−^ KO mice and their WT littermate controls.

The mice were subjected to OIR and sacrificed at various time points to examine the effect of A1 deletion on the vascular injury, vascular repair and pathological RNV.

**Fig. 4:**
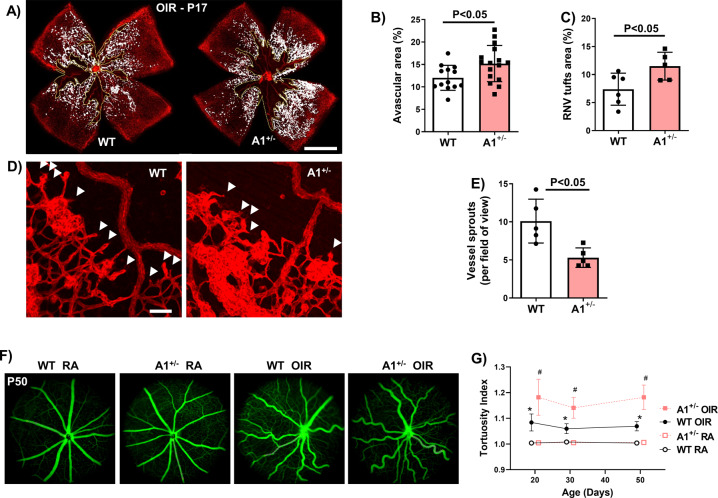
A1 deletion increases pathological neovascularization and decreases vascular repair at P17. **A**–**C** WT and A1 KO littermates were subjected to OIR at P7 and prepared for analysis on P17. Avascular area (AVA, yellow outline) and pathological RNV (tufts, highlighted in white) were significantly increased in A1 KO retinas. Scale bar = 100 µm. **D**, **E** Higher magnification images show decreased vessel sprouts at the AVA border zone of the P17 lectin-labeled A1 KO retina flatmounts. Scale bar = 20 µm. **F**, **G** Fluorescein angiography images show persistent vessel tortuosity in the WT OIR retinas through P50, which was increased in the A1 KO retinas. Data are presented as mean ± SD in all the figures.

Morphometric analyses of isolectin B4-labeled retinal flatmounts prepared from A1 KO mice exposed to hyperoxia from P7 to P12 and returned to normoxia from P12 to P17, showed significant increases in both AVA and pathological RNV tuft formation as compared to the WT littermate controls (Fig. 4A–C). Moreover, these alterations were accompanied by significant decreases in vascular sprouting and tip cell formation in the A1 KO retinas (Fig. 4D, E**)**. Further analysis of retinal flatmounts prepared after 2 days of hyperoxia, at the peak time of vaso-obliteration, showed no effect of the A1 KO on vaso-obliteration (Fig. S3A–C). Of note, the A1 but not A2 mRNA expression was reduced by ~50% in the A1^+/−^ KO retinas under RA confirming hemizygous deletion of A1 (Fig. S3D, S3E). Taken together, these results suggest that A1 expression is not involved in hyperoxia-induced vaso-obliteration but does play a role in promoting vascular repair and limiting pathological RNV after the hyperoxia-induced injury.

Analysis of vascular tortuosity following the OIR exposure using fluorescein angiography showed persistent vascular tortuosity on P50 (Fig. 4F, G, Fig. S3F). This damage was worsened in the A1 KO mice.

### Global A1 deletion worsens OIR-induced retinal injury

**Fig. 5:**
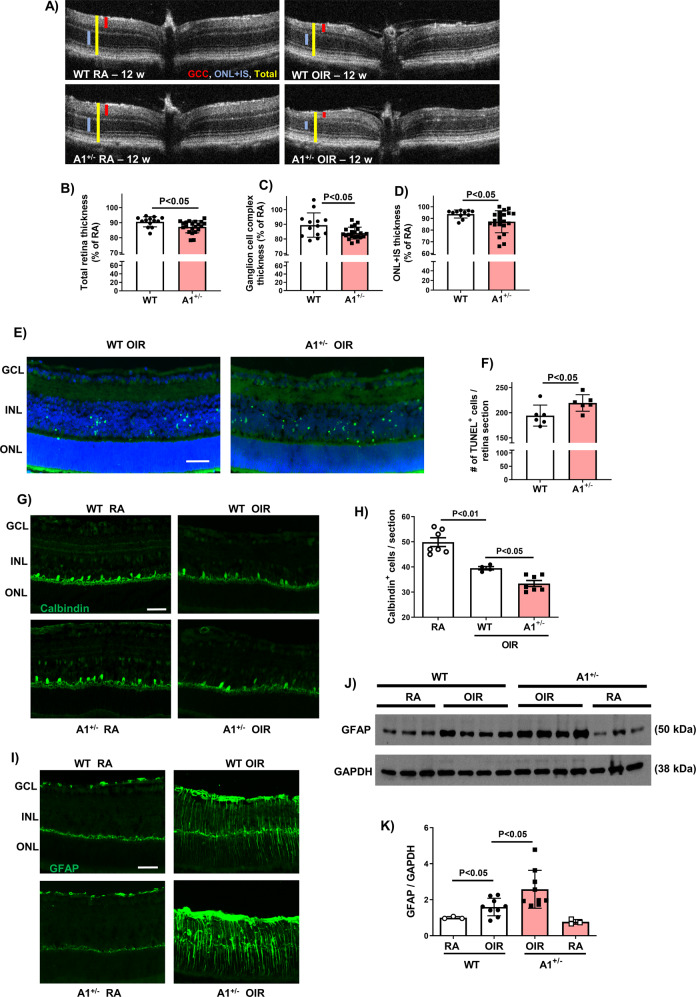
A1 deletion worsens retinal thinning, apoptosis, horizontal cells loss, and glial activation after OIR. **A**–**D** SD-OCT analysis of 12-week-old mice retinas showed a significant reduction in thickness of the total retina, ganglion cell complex (GCC), and outer nuclear layer plus inner segments (ONL + IS) in the A1 KO OIR retinas as compared to WT OIR retinas. **E**, **F** TUNEL labeling and quantification at P9 showed a significant increase in TUNEL-positive cells in A1 KO OIR retina sections as compared to WT OIR retinas. Scale bar = 50 µm. **G**, **H** Calbindin labeling and quantitation of retina sections at P17 showed horizontal cell loss after OIR that was further aggravated in A1 KO OIR retinas. Scale bar = 50 µm. **I**–**K** GFAP immunolabeling of retina cross-sections at P17 showed increased expression of GFAP in Müller cells beyond their end-feet after OIR, which was further increased in A1 KO retinas. Western blotting on P17 retina homogenates and quantification confirmed the immunolabeling results. Scale bar = 50 µm.

Chronic morphological changes and retinal thinning have been previously reported in mice subjected to OIR [26]. We determined retinal thickness in WT and A1 KO mice subjected to OIR by using spectral domain optical coherence tomography (SD-OCT). This analysis showed a significant worsening of retinal thinning in A1 KO mice as compared to the WT OIR group. This was evidenced by decreases in thickness of the total retina, ganglion cell complex (GCC) and outer nuclear layer plus inner segments (ONL + IS) (Fig. 5A–D). Thickness of the various retinal layers was not different among the RA groups (Fig. S3G–I).

The worsening of OIR-induced retinal thinning in the A1 KO retina was preceded by increases in neuronal death as shown by a rise in TUNEL-positive cells (Fig. 5E, F) and a decrease in numbers of horizontal cells (Fig. 5G, H, Fig. S3J) in the A1 KO OIR retinas as compared with their WT littermates. Furthermore, A1 KO OIR retinas showed increased glial activation as measured by GFAP immunolabeling and western blotting (Fig. 5I–K).

### A1 is increased in mouse and human RNV conditions

**Fig. 6:**
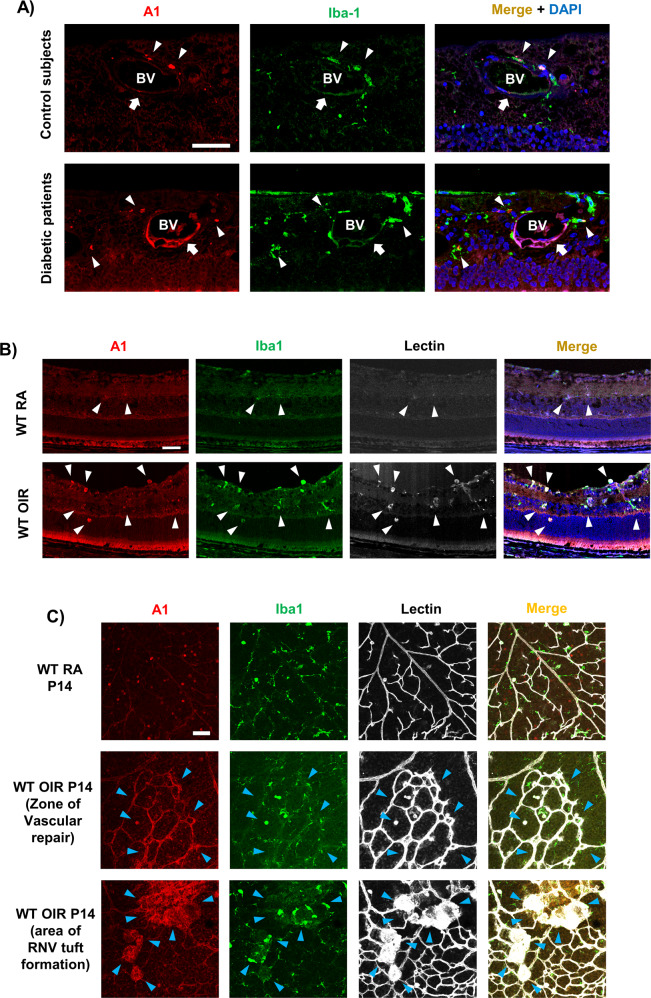
A1 is upregulated in human DR and mouse OIR retinal sections. **A** Immunolabeling of retina sections from human donor with PDR showed increased co-localization of A1 with Iba-1 and blood vessel (BV)-like structures as compared with non-diabetic control. Scale bar = 50 µm. **B** Immunolabeling of P17 mouse retina sections showed co-localization of A1 with Iba-1-positive myeloid cells (microglia/macrophages) and lectin-positive blood vessels after OIR. Scale bar = 20 µm. **C** Immunolabeling of mouse retina flatmount at P14 showed A1 upregulation and co-localization with Iba-1-positive microglia/macrophages and lectin-positive blood vessels in both the areas of vascular repair (VR) and RNV tufts (RNV). Scale bar = 20 µm.

We next examined A1 expression and co-localization in human and mouse retinas. Immunolabeling on postmortem human retina sections from patients with proliferative diabetic retinopathy showed increased levels of A1 immunoreactivity with Iba-1-positive microglia/macrophages and vessel-like structures as compared to sections from non-diabetic controls (Fig. 6A). Similarly, immunolabeling studies of the mouse retinas during the RNV phase of OIR showed co-localization of A1 with blood vessels as well as microglia/macrophages in retina sections (Fig. 6B) and flatmounts (Fig. 6C).

To gain insight on how amino acids and polyamines involved in the arginase pathway changed in the OIR retinas in response to A1 treatment or deletion, we performed an LC-MS analysis of retina lysates. Arginine, ornithine, putrescine, and citrulline levels were higher in the A1 KO OIR retinas as compared with the WT OIR retinas. To our surprise, PEG-A1 did not alter levels of arginine, ornithine, or citrulline as compared with the WT OIR retinas while putrescine, which increased in the A1KO OIR retinas was decreased with PEG-A1 treatment (Fig. S4). It appears that other pathways of ornithine/putrescine formation are altered with A1 deletion and/or PEG-A1 treatment leading to these counterintuitive results.

We further assessed A1 and A2 mRNA levels in blood samples from infants with or without ROP. A1, but not A2, was increased in ROP samples as compared to controls yet this did not reach statistical significance (Fig. S5A, B). Furthermore, there was a trend towards an increase in arginase activity in vitrectomy samples from patients with diabetic retinopathy (DR) as compared to patients without DR (Fig. S5C).

### Neither endothelial nor myeloid A1 protect against OIR-induced retinal injury

**Fig. 7:**
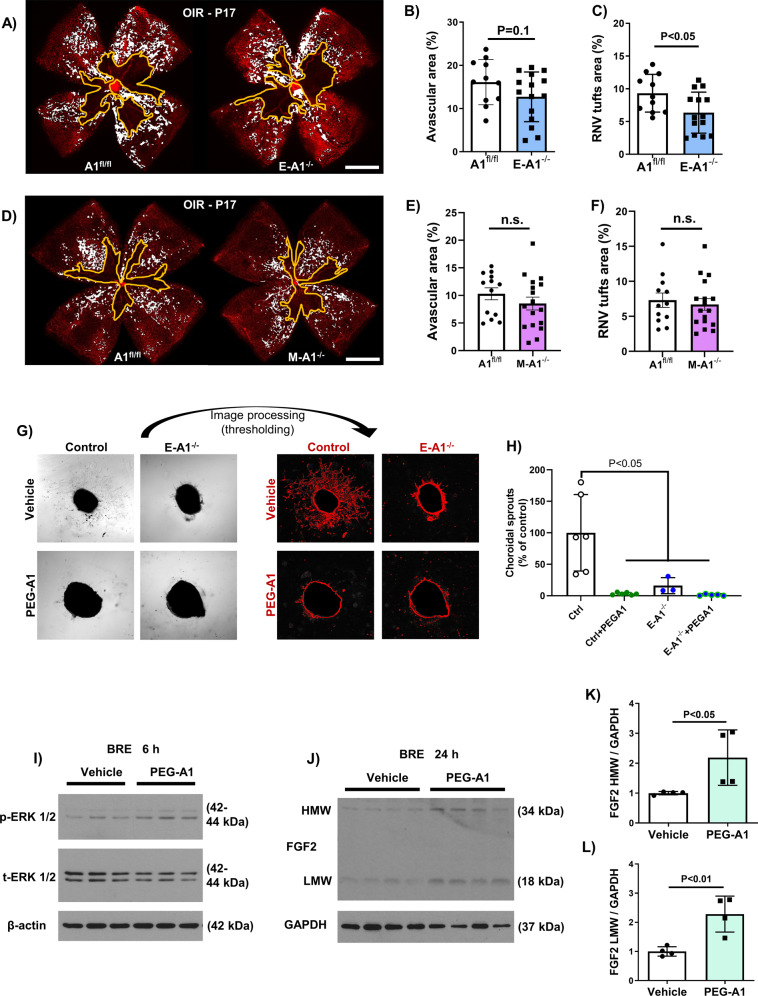
Endothelial but not myeloid A1 deletion decreases pathological angiogenesis in OIR. **A**–**C** Analysis of P17 OIR retinas or using lectin staining showed a significant decrease in pathological neovascularization (NV, highlighted in white) in endothelial A1 knockout mice (E-A1^−/−^) as compared to littermate floxed controls (A1^f/f^), while the AVA (yellow outline) showed a decreasing trend that was not statistically significant. Scale bar = 100 µm. **D**–**F** Analysis of myeloid A1 knockout mice (M-A1^−/−^) OIR retinas showed no change in AVA or NV tuft formation at P17 as compared to littermate floxed controls (A1^f/f^). Scale bar = 100 µm. **G**, **H** Choroidal angiogenesis assay and quantification showed marked suppression of the angiogenic response with endothelial A1 deletion or PEG-A1 treatment. **I**–**L** Treatment of bovine retinal endothelial cells with PEG-A1 for 6 h led to increased ERK phosphorylation as measured by western blotting while 24 h treatment increased FGF2 protein levels.

Based on the immunolocalization data showing expression of A1 in areas of RNV and associated macrophages/microglia, we utilized mice lacking A1 in endothelial or myeloid cells using Cdh5 and LysM cre promoters, respectively. Mice lacking A1 in endothelial cells exhibited a significant reduction in RNV tuft formation at P17, while the AVA showed a trend towards improved repair that was not statistically significant (Fig. 7A–C). On the other hand, myeloid-specific A1 deletion had no effect on either AVA or RNV (Fig. 7D–F). Since the protective effect of endothelial A1 deletion in limiting RNV contradicts the beneficial actions of A1 supplementation with PEG-A1, we further confirmed our results using a model of ex vivo choroidal angiogenesis. This uncontrolled vessel sprouting assay was performed in complete media using choroid explants from control and endothelial A1 KO mice. Confirming the pathological RNV data in vivo, deletion of endogenous endothelial A1 or exogenous PEG-A1 treatment reduced the choroidal vessel sprouting (Fig. 7G, H). Moreover, treatment of bovine retinal endothelial cells (BRE) with PEG-A1 increased ERK phosphorylation and FGF2 protein expression indicating a direct effect of PEG-A1 on triggering retinal vascular expression of FGF2 (Fig. 7I–L). Collectively, these data suggest a cell-specific effect of A1 in retinal angiogenesis during OIR.

## Discussion

Our studies in models of acute retinal and brain injury have shown that treatment with a stable, pegylated drug form of recombinant A1 protein limits neuronal injury [13]. Here, we show a protective function of A1 in limiting vascular and neuronal injury in the OIR mouse model. Treatment with pegylated recombinant A1 protein enhanced vascular repair, inhibited pathological RNV, and reduced vascular tortuosity, all of which were associated with preservation of retinal function. Deletion of one copy of the A1 gene worsened pathological RNV and increased retinal injury, confirming the critical role of A1 in limiting retinal injury.

In addition to its adverse effects on the retinal vasculature, OIR also induces retinal neuro-glial injury. Our previous studies have shown OIR-induced damage of multiple cell types including horizontal cells, amacrine cells, bipolar cells, Müller glia and photoreceptors [6, 7]. In this study, we found that A1 treatment reduced production of the apoptotic marker, cleaved PARP and rescued the horizontal cell loss after OIR. In contrast, A1 deletion exacerbated the OIR-induced injury as indicated by increases in TUNEL labeling and decreases in horizontal cell survival. While pathological RNV in the OIR model is normalized by P25, vascular tortuosity and functional deficits persist thereafter [27, 28]. Our study showed that A1 treatment significantly reduced late-stage defects in vascular tortuosity and improved visual acuity in OIR mice examined after they reached adulthood. In contrast, A1 deletion significantly worsened the vascular tortuosity and exacerbated the OIR-induced retinal thinning, indicating the sustained detrimental effects of the hemizygous deletion of the A1 gene.

PEG-A1 is an investigational drug that is currently being tested in clinical trials of certain types of cancers that are dependent on l-arginine. PEG-A1 has been shown to be safe and well-tolerated in humans, which further makes this pathway a promising target for different disease conditions. We have previously shown the PEG-A1 is neuroprotective in the retinal ischemia-reperfusion injury model [11]. After intravitreal injection of PEG-A1, arginase activity remained high in the mouse vitreous for at least three days making it a suitable treatment for eye disorders because of its favorable pharmacokinetic profile. Our recent studies in models of ischemia/reperfusion injury, optic nerve crush, and ischemic stroke have shown protective efficacy of systemic drug delivery [13]. Further investigations will be needed to assess relative merits vs risks of intravitreal vs systemic drug delivery.

PEG-A1 treatment dampened the inflammatory response during the hypoxia phase of OIR. Cytokines and growth factors including TNFα, IL-6, MCP1, and VEGF are upregulated in the vitreous of patients with proliferative retinopathy [29–32], and have been shown to mediate pathological neovascularization in OIR [33–35]. PEG-A1 treatment decreased the expression of these pro-angiogenic factors. PEG-A1 treatment increased the expression of A1 mRNA. The PEG-A1-induced increase in A1 expression is consistent with the increase in macrophage/microglial cells that were both strongly positive for the M2-like macrophage-microglia marker CD206 and the M1-like marker CD16/32. This double-positive M1/M2-like phenotype is characteristic of the angiogenic phenotype seen in our previous study using the OIR model. Our previous study has shown a massive (60-fold) upregulation of A1 mRNA in microglia/macrophages during RNV in the OIR model, which was the highest increase among the genes examined with RNAseq [36]. Moreover, the increase in A1 in microglia/macrophages was associated with FGF2 upregulation. Our current work shows a strong increase in F4/80, FGF2 double-positive microglia/macrophages in the zone of increased tip cell formation and around the resolving RNV following PEG-A1 treatment. This may be reflective of their transition to a reparative phenotype. Further work is required to investigate this possibility.

Surprisingly, mice with myeloid cell-specific deletion of A1 did not show any differences in vaso-obliteration or neovascularization as compared with their littermate controls. The fact that A1 expression in endothelial or myeloid cells is dispensable for the protective effect of A1 suggests that microglial A1 expression and/or systemic/circulating A1 may be mediating the protective phenotype. Future studies will be conducted to confirm this hypothesis.

In addition to increasing A1 expression, the PEG-A1 treatment dampened the OIR-induced upregulation of the inducible NOS enzyme iNOS. Activity of iNOS, which plays a crucial role in the inflammatory process and pathological neovascularization, induces retinal thinning and increases apoptosis [37, 38]. The protective effects of PEG-A1 on iNOS could be due to the above suggested actions in promoting a reparative macrophage/microglial phenotype, but could also be due to the actions of A1 in limiting arginine availability. Studies have shown that extracellular arginine deprivation or intracellular arginase overexpression leads to decreases in both expression and activity of iNOS [39]. Moreover, the A1-induced depression in iNOS-derived NO formation could be responsible for the associated decrease in VEGF expression in that NO is known to increase VEGF expression via activation of hypoxia inducible factor 1 [39, 40].

In addition to the above effect of reducing iNOS and VEGF expression, PEG-A1 increased the levels of FGF2 and CNTF. Both growth factors have been shown to facilitate physiological revascularization and reduce pathological RNV [24, 25]. These results show that PEG-A1 improves OIR outcome by dampening the retinal inflammatory response, decreasing iNOS and VEGF expression and upregulating neurotrophic factors. Our further studies using cultured retinal endothelial cells showed that PEG-A1 can directly stimulate their expression of FGF2 protein, possibly through ERK activation [41–43]. In line with this, previous studies have shown that depletion of l-arginine leads to ERK metabolic reprograming [44]. However, this effect on endothelial cells appears to be mediated by exogenous rather than intracellular A1 since endothelial-specific deletion of A1 unexpectedly reduced RNV tuft formation in vivo, which we further confirmed by an ex vivo assay of uncontrolled angiogenesis in choroidal explants. It is possible that the endothelial cell-specific A1 deletion suppresses angiogenesis by reducing formation of the A1 products, polyamines, and proline, which are required for endothelial cell growth and collagen formation, respectively [45, 46].

Our studies of the retina amino acids and polyamines involved in the arginase pathway showed an increase in arginine, citrulline, ornithine, and putrescine in the A1 KO after OIR, while PEG-A1 treatment did not alter arginine, citrulline, or ornithine and decreased putrescine. The increases in arginine and citrulline in the A1 KO retina are consistent with the decrease in A1 expression, which would increase arginine availability to NOS, leading to increased formation of NO and citrulline. However, the increase in ornithine and putrescine in the A1 KO retina is counterintuitive since ornithine and putrescine are down stream of arginase. It is possible that arginase modulation in the OIR retina disturbs other enzymes that play a role in ornithine and putrescine production such as arginine decarboxylase that converts arginine to putrescine via agmatine and/or ornithine amino transferase, which is an alternative route to ornithine and polyamine formation via the glutamine/glutamate pathway (Supplementary Fig. 4O). The significance of these changes in the OIR retina is yet to be elucidated. However, studies in models of ischemic injury have shown that polyamines, especially putrescine, play a role in excitotoxic injury via activation of the NMDA (n-methyl-d-aspartate) receptor [47]. Thus, the decrease in putrescine after PEG-A1 treatment is consistent with its action in limiting OIR-induced neurovascular injury.

In conclusion, A1 has protective actions in the OIR model by enhancing reparative angiogenesis and promoting neuronal survival. We have previously shown that additional deletion of one copy of A1 in A2^−/−^ mice suppressed the vascular normalization and protection conferred by A2 deletion [8]. Taken together with our findings in this study, it is possible that the protection seen with A2 deletion in OIR is at least partially mediated through upregulation of A1. PEG-A1 represents a novel therapy to target neurovascular injury in ROP and other forms of ischemic retinopathy.

Our detailed examination of the OIR vascular, neuronal and functional phenotypes in pups and young adult mice combined with a robust sample size and blinded analysis confirmed a major role of A1 in this model. We also report upregulation of A1 in human ischemic retinopathy disease conditions, which further translates our findings from bench to bedside and strongly suggest an important role of A1 in human retinal pathology.

## Supplementary information

Supplementary file

Reporting summary

## Data Availability

All data generated in this study are included in this published article and its supplementary information files.
