# Supplementary material for: Targeting proliferative retinopathy: Arginase 1 limits vitreoretinal neovascularization and promotes angiogenic repair
Source: Cell Death Dis. 2022 Aug 29;13(8):745. doi: 10.1038/s41419-022-05196-8 (PMC9424300; doi:10.1038/s41419-022-05196-8)
Supplement: Supplementary file 1 — Supplementary file [file 41419_2022_5196_MOESM1_ESM.pdf]

Targeting proliferative retinopathy:

Arginase 1 limits vitreoretinal neovascularization and promotes angiogenic repair

DETAILED METHODS:

Human samples

Infants with or without ROP: Blood Samples were collected from premature infants after guardian consent patients and processed for RT-PCR analysis. Patient information is provided in **supplementary table 1**.

Vitrectomy samples: Samples were collected from patients undergoing pars plana vitrectomy (PPV) due to proliferative diabetic retinopathy (PDR) with retinal detachment and patients undergoing PPV for reasons other than PDR (chronic retinal detachment, open globe injury, or macular hole). Vitrectomy samples were concentrated using Amicon Ultra 30k centrifuge filter devices (cat # UFC903008) and arginase activity was measured as previously described.(1) Patient information is provided in **supplementary table 2**.

Human retina sections: Sections from patients with proliferative diabetic retinopathy (PDR) and age-matched non-diabetic controls were purchased from National Disease Research Interchange (NDRI, Philadelphia, PA). Sections were deparaffinized and immunolabeled as described under ‘immunofluorescence labeling’ section. Patient information is provided in **supplementary table 3**.

Analysis of vaso-obliteration and neovascularization

Eyeballs were fixed in 4% paraformaldehyde (PFA) overnight. Retina flatmounts were dissected and then blocked and permeabilized in phosphate-buffered saline (PBS) containing 10% goat serum and 1% Triton X-100 (Sigma-Aldrich) for 30 minutes. Retinas were then immunolabeled with Alex594-labeled *Griffonia simplicifolia* isolectin B4 (1:200; Invitrogen, Carlsbad, CA) overnight at 4°C. Retinas were flatmounted in mounting medium (Vectashield; Vector Laboratories, Burlingame, CA) and images were captured using fluorescence microscopy (Axioplan 2, Carl Zeiss Vision Inc.). Areas of central capillary dropout (avascular area, AVA) and pathological RNV tufts were quantified in a masked fashion using ImageJ software (NIH, Bethesda, MD).(2, 3) High-magnification images were acquired to perform vessel sprouting analysis. Vessel sprouts were counted as previously described.(3)

Analysis of retinal vessel tortuosity

Retinal vessel tortuosity was measured using fluorescein angiography. Mice were anesthetized with ketamine/xylazine cocktail (i.p.), pupils were dilated with 1% tropicamide (Akorn, Lake Forest, IL, USA) and corneas were moisturized with GenTeal Systane lubricant eye drops (Alcon, Ft. Worth, TX). Fluorescein injection solution (AK-Fluor 10%, Akorn) was injected i.p. and retinas were imaged using the Micron IV system (Phoenix Laboratories). Vessel tortuosity was quantified using customized MATLAB software (www.quantbv.com) as previously described.[(4)] Briefly, for measuring vessel tortuosity, we used a cursor to select branch points along the artery beginning from the optic nerve to a distance of 275±25 µm. A linear projection was then drawn to connect the first and last points automatically. Tortuosity was calculated as the ratio of actual vessel length to the projected linear length. Values of 1 represented complete lack of tortuosity, and values greater than 1 represented the extent of tortuosity.

Immunofluorescence labeling

PFA fixed eyeballs were washed in PBS and cryoprotected. Cryostat sections (10 µm) were permeabilized in 1% Triton (20 min) and blocked in 10% normal goat serum containing 1% BSA (one hour). Sections were then incubated overnight in primary antibodies at 4°C. On day two, the sections were incubated at room temperature for 1 hour in fluorescent conjugated secondary antibodies (Life Technologies), washed in PBS and mounted with Vectashield (Vector Laboratories). The following primary antibodies were used: GFAP (Dako, cat. # Z0334), calbindin (Sigma, cat. # C9848), Iba-1 (Wako, cat. # 019-19741), A1 (Sigma-Aldrich, cat. # ABS535), F4/80 (Abcam, cat. # ab6640), FGF2 (Abcam, cat. # ab8880), CD16/32, (BD Biosciences, cat. # 553142), and CD206 (Abcam, cat. # ab64693). Quantification of positive cells was performed as previously described.(5)

Quantitative Real-Time PCR

Extraction of retinal total RNA and reverse-transcription procedures were performed as described previously.(6) Quantitative PCR was performed using ABI StepOne Plus Thermocycler (Applied Biosystems, Foster City, CA, USA). The probes of TaqMan assays (Invitrogen, Carlsbad, CA, USA) used to detect mouse A1, A2, Ciliary Neurotrophic Factor (CNTF), Fibroblast Growth Factor 2 (FGF2), interleukin 6 (IL-6) and hypoxanthine phosphoribosyltransferase (HPRT) as internal control were Mm00475988_m1, Mm00477592_m1, Mm00446373_m1, Mm01285715_m1, Mm00446190_m1 and Mm00446968_m1, respectively. Other primer sequences for mouse transcripts are provided in **supplementary table 4**. Data analysis was performed using $\Delta\Delta C_T$ method as described previously.(6)

Western blotting analysis

Retinal lysates were homogenized in RIPA buffer supplemented with protease and phosphatase inhibitors. Samples (20 µg protein) were run using 10% sodium dodecyl sulfate-polyacrylamide gel electrophoresis, transferred to nitrocellulose membrane and reacted with primary antibody followed by horseradish peroxidase-linked secondary antibody and enhanced chemiluminescence (Amersham Pharmacia). β-actin (Sigma-Aldrich, cat. # A5441) or GAPDH (Meridian Bioscience, cat. # H86504M) served as a loading control. The following primary antibodies were used: GFAP (Sigma, cat. # G6171), PARP (Cell signaling, cat. # 9542S), A1 (Sigma-Aldrich, cat. # ABS535), FGF2 (Abcam, cat. # ab8880), ERK (Cell signaling, cat. # 4695S), p-ERK (Cell signaling, cat. # 4370S), A2 (Santa Cruz, cat. # Sc-20151), iNOS (Cell signaling, cat. # 13120) and VEGF (Abcam, cat. # ab46154). un-cropped images of the original western blots from which figures have been derived are shown in **fig. S5**.

TUNEL assay

TUNEL assay was performed on retinal sections using TdT-mediated dUTP nick end labeling (In Situ Cell Death Detection Kit, Roche) to detect apoptotic cells based on the manufacturer recommendations. Counting and quantification was performed as previously described.(7)

OptoMotry

OptoMotry (Cerebral Mechanics Inc.) was employed for screening visual function using the opto-kinetic tracking (OKT) response.(8, 9) Individual unrestrained mice were placed on an elevated platform surrounded by four computer monitors. The monitors project a virtual stimulus in the form of a sine wave that rotates around the animal. Animals track the grating with reflexive head and neck movements in the direction of grid rotation. By reversing the direction of the grid rotation, the system

measures visual function in each eye separately because motion in the temporal to nasal direction of either eye elicits a tracking response. A camera monitors the behavior of the animal from above, allowing the rater to detect the mouse tracking responses in real time and give a score of yes or no. The whole study was conducted by one rater who was blinded to the treatment groups.

To examine visual acuity, spatial frequency thresholds (cycles per degree) were measured by systematically increasing the spatial frequency of the grating (decreasing the bar width) at full contrast until mice no longer tracked. Rotation speed was fixed at 12 degrees per second and the data was managed and generated by the software. Data are presented as responses from both eyes.

Spectral Domain Optical Coherence Tomography (SD-OCT)

SD-OCT was performed to investigate the integrity of the retinal layers. Briefly, mice were anesthetized, and pupils were dilated with 1% tropicamide (Bausch & Lomb, Tampa, FL, USA). Corneas were then moisturized with GenTeal Lubricant Eye Gel (Alcon, Ft. Worth, TX) and Systane lubricant eye drops (Alcon) were applied. The Bioptigen Spectral-Domain Ophthalmic Imaging System (Bioptigen, Envisu R2200, Morrisville, NC, USA) was used to obtain live images from the mice. Imaging included averaged single B scan and volume intensity scans (VIP) with images centered on the optic nerve head. The auto segmentation report of analysis in all retinal layers was processed using InVivoVue Diver 2.4 software (Bioptigen). This measured thickness of the total retina along with that of the different retinal layers.

Electroretinogram (ERG)

ERG was performed to investigate neuronal functions using the Celeris-Diagnosys system (Diagnosys, Lowell, MA, USA). After overnight dark adaptation, mice were anesthetized and pupils were dilated with topical 0.5% tropicamide (Akorn, Lake Forest, IL, USA) and 2.5% phenylephrine HCL (Paragon BioTeck, Portland, OR, USA). Mouse corneas were then moisturized with a thin layer of GenTeal Lubricant Eye Gel (Alcon, Ft. Worth, TX). The light guide electrodes were placed on the gel and recording began after the scotopic and photopic testing was set up. For each animal, the light guide electrode was used to present light flashes over a range of intensities and record ERG responses. The a-wave amplitude was measured as the difference between the pre-stimulus baseline and the trough of the a-wave. The b-wave amplitude was measured from the trough of the a-wave to the peak of the b-wave. The results are presented as averaged values of amplitude with the two eyes of each mouse.

Endothelial cell culture

Bovine retinal endothelial cells (BRE) from passages 4-8 were used for the cell culture experiments. BREs were isolated by our group as previously described.(10) Cells were grown in M199 media containing 10% fetal bovine serum (FBS), 1% Penicillin/Streptomycin (Gimini, West Sacramento, CA) and 10% cell systems complete media. Cells were shifted to serum starvation M199 media containing 0.2% FBS and 0.1% bovine serum albumin (BSA) overnight before PEG-A1 treatment (1 µg/ml or PBS) for 6 or 24 hours. Cells were then collected for western blotting.

Choroidal angiogenesis assay:

Choroidal angiogenesis was examined based on previously published protocols with minor modifications.(11, 12) Mice were euthanized under deep anesthesia and eyeballs were collects.

Cornea and lens were dissected out and removed under a dissection microscope and choroid-scleral complex was separated from the retina. Choroidal punches of 1 mm diameter were taken using 1 mm biopsy punches with plunger (Integra Miltex). Punches were transferred into the center of 30 µL Matrigel dome in 48-well plate. Choroidal explants were incubated in CSC complete medium (Cell Systems) supplemented with FBS, and media was changed every other day. The assay was conducted in complete media to examine uncontrolled angiogenesis. PEG-A1 treatment (1 µg/ml or PBS) started from the beginning of the assay and was added with every media change. Sprouting area was quantified using ImageJ software.

Sample preparation for liquid chromatography-tandem mass spectrometry with multiple reaction monitoring (LC-MRM MS) analysis

Eighty µL 80% acetonitrile was added to each mouse retinal sample together with 100 µL stainless steel beads blend (SSB14B, Next Advance). The mixture tube was beaten in Bullet Blender (BBX24, Next Advance) with speed 8 and time 3 at 4 °C. The sample was then centrifuged at 16,000 g for 15 minutes and 50 µL of the supernatant was transferred into a new tube for derivatization with tosyl chloride.

Tosyl chloride derivatization was performed by mixing 50 µL of retinal sample supernatant with 50 µL tosyl chloride solution (10 mg/mL in acetonitrile) and 25 µL borate buffer (0.5M, pH=11). After mixing, samples were incubated for 2 hours at 50 °C in water bath.

LC-MRM MS analysis

Sample separation was performed using a Phenomenex Kinetex C18 column (100x2.1mm, 1.7um) on a Shimadzu Nexera UHPLC system at a flowrate of 0.2 mL/min using gradient elution from 10% to 95% acetonitrile (with 0.1% formic acid) in 6 minutes. The effluent was ionized using positive ion electrospray on a TSQ Quantiva triple-quadrupole mass spectrometry with the following instrument settings: ion spray voltage 3500V, sheath gas 10, ion transfer tube temperature 350, aux gas 5, and Q1/Q3 resolution of 0.7 FWHM. The optimal collision energy and RF lens were determined using purchased standards. The transitions monitored are listed in the following table.

Compound	Polarity	Precursor (m/z)	Product (m/z)	Collision Energy (V)
Put_dev_tosyl_397(+1)	Positive	397.11	226.058	15.8
Spd_dev_tosyl_3T(+1)	Positive	608.28	226.058	23
Spm_dev_tosyl_4T(+1)	Positive	819.336	437.183	30.1
Cit_dev_tosyl(+1)	Positive	330.1	313	13.6
Arg_dev_tosyl(+1)	Positive	329.2	224	13.5
Orn_dev_tosyl_2T(+1)	Positive	441.1	224.058	17.3
Pro_dev_tosyl(+1)	Positive	270.1	224	11.5

The integrated peak areas for these transitions were calculated for each sample using Skyline software (version 20.0, University of Washington).

SUPPLEMENTARY TABLES:

Table 1: Premature infants' information		
**Parameter**	**Control infants**	**ROP infants**
Number [sex]	4 [3 ♂, 1 ♀]	4 [4 ♂, 0 ♀]
Gestational Age (GA) in weeks [SD]	26 [1]	24 [1]
Postconceptional age PCA in weeks [SD]	35 [3/7]	36 5/7 [3 2/7]
Body weight in grams [SD]	595 [194]	663 [75]
Disease (ROP) stage*<br>[# of patients with condition]	-	Z1/S3 [1]<br>Z1/S3+ [1]<br>Z2/S3+ [2]
Comorbidities* [# of patients with condition]	BDP [3]<br>BPD ± PDA [1]	BDP [2]<br>BPD ± PDA [2]

*zone (Z) 1-3 and stage (S) 1-5 with plus disease (+), Patent ductus arteriosus (PDA), Bronchopulmonary dysplasia (BPD).

Table 2: Human vitreous samples information		
**Parameter**	**Non-diabetic patients**	**Diabetic patients**
Number [Sex]	3 [1 ♂, 2 ♀]	4 [1 ♂, 3 ♀]
Race [number]	Black [3]	White [1], Black [3]
Age in years [SD]	50 [28]	49 [20]
Diabetes status [number]	Non-diabetic	Type 1 diabetes [1] or type 2 diabetes [3]
HbA1c [SD]	NA	8.8 [1.2]
Reasons for PPV	Chronic retinal detachment, Open globe injury, or macular hole	Proliferative diabetic retinopathy (PDR) + tractional retinal detachment (TDR)

Table 3: Human retina sections information		
**Parameter**	**Control subjects**	**Diabetic patients**
Number [Sex]	3 [2 ♂, 1 ♀]	3 [2 ♂, 1 ♀]
Race	White	White
Age in years [SD]	69 [9]	70 [8]
Diabetic retinopathy status	No diabetic retinopathy	Proliferative diabetic retinopathy (PDR)

Table 4: RT-PCR primers sequences		
**Gene Name**	**Forward Primer**	**Reverse Primer**
TNFα	GGTCCCCAAAGGGATGAGAA	TGAGGGTCTGGGCCATAGAA
MCP1	GGCTCAGCCAGATGCAGTTAA	CCTACTCATTGGGATCATCTTGCT
iNOS	GGCAGCCTGTGAGACCTTTG	TGCATTGGAAGTGAAGCGTTT
VEGF	TACCTCCACCATGCCAAGTG	TCATGGGACTTCTGCTCTCCTT
HPRT	TCAAAGTGCCAGTGAACCCC	GGTCACAGCCAGTCCTCTTAC

SUPPLEMENTARY FIGURES:

S1

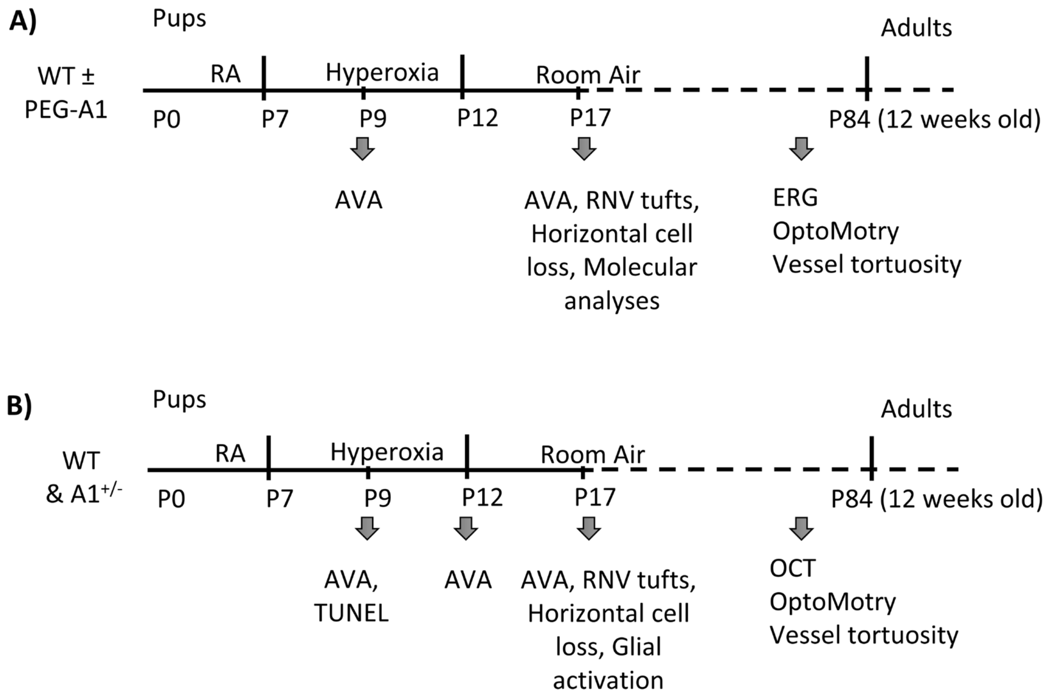

Fig. S1: Schematic representation of the experimental groups, treatments and time-points for the analyses.

(A) WT pups were treated with PEG-A1 or vehicle and sacrificed at the vaso-obliteration phase, the neovascularization phase, or at later stages of adulthood.

(B) Similarly, WT and $A1^{+/-}$ pups were subjected to OIR at P7 and sacrificed during the vaso-obliteration phase, the neovascularization phase, or at later stages of adulthood.

S2

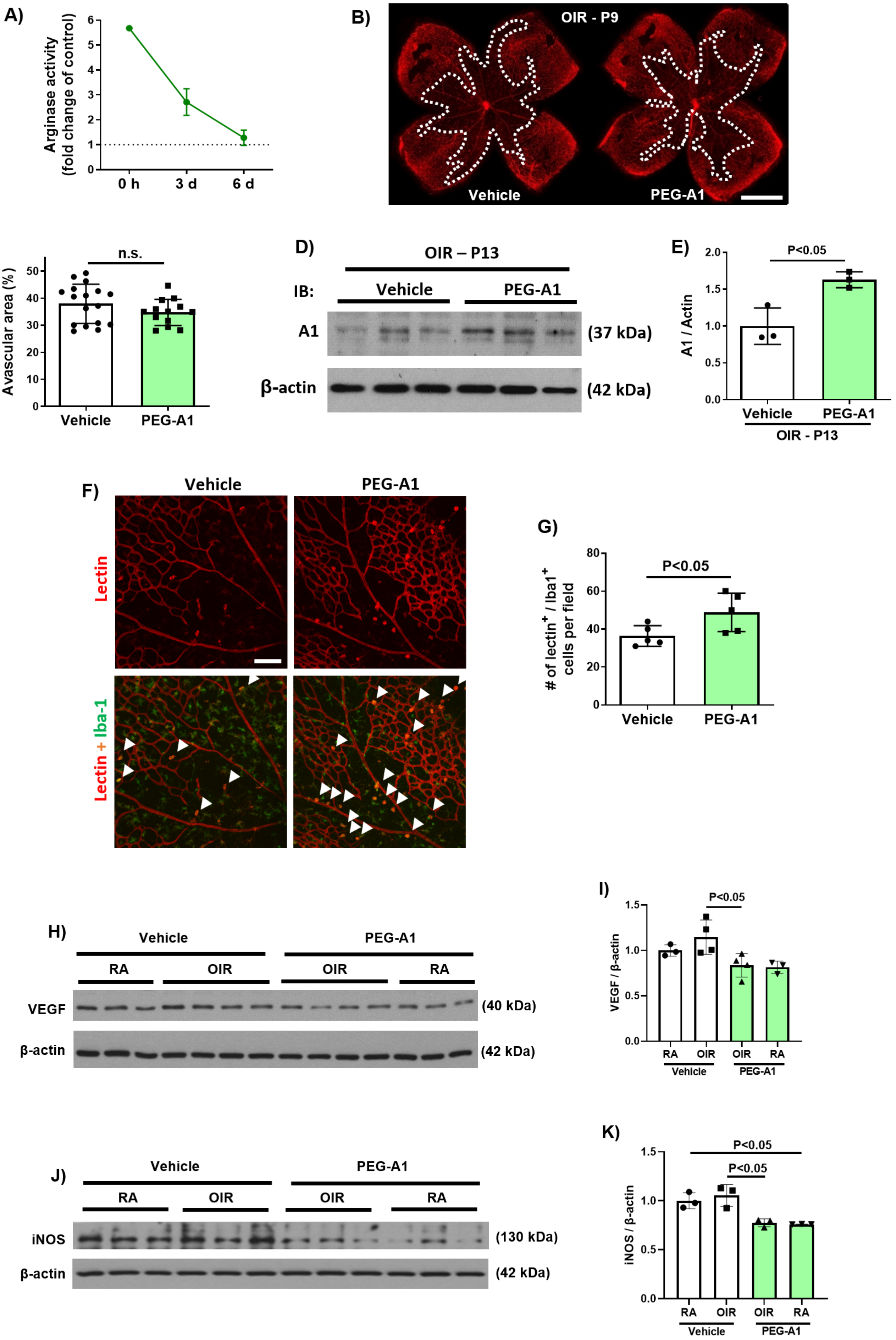
A)
Arginase activity (fold change of control)
0 h
3 d
6 d
B)
OIR - P9
Vehicle
PEG-A1
C)
n.s.
Avascular area (%)
Vehicle
PEG-A1
D)
OIR – P13
IB:
Vehicle
PEG-A1
A1
(37 kDa)
β-actin
(42 kDa)
E)
P<0.05
A1 / Actin
Vehicle
PEG-A1
OIR - P13
F)
Vehicle
PEG-A1
Lectin
Lectin + Iba-1
G)
P<0.05
# of lectin+ / Iba1+ cells per field
Vehicle
PEG-A1
H)
Vehicle
PEG-A1
RA
OIR
OIR
RA
VEGF
(40 kDa)
β-actin
(42 kDa)
I)
P<0.05
VEGF / β-actin
RA
OIR
OIR
RA
Vehicle
PEG-A1
J)
Vehicle
PEG-A1
RA
OIR
OIR
RA
iNOS
(130 kDa)
β-actin
(42 kDa)
K)
P<0.05
P<0.05
iNOS / β-actin
RA
OIR
OIR
RA
Vehicle
PEG-A1

Fig. S2: Supporting data from WT mice treated with PEG-A1.

(A) Analysis of arginase activity in mouse vitreous after PEG-A1 intravitreal injection as compared to basal arginase activity in vehicle injected eyes showed ~ 6-fold increase in activity after injection (0 hour) which decreased to 3-fold at day 3 after injection and further decreased at day 6. Vitreous was collected by removing the vitreous body together with the retina and lens and then placing them in centrifuge tube filters with 50 µl of PBS followed by immediate spinning at high speed to separate the vitreous and PBS from the retina and lens. Arginase activity was measured as described previously.(1)

(B, C) WT mice were treated with a single intravitreal PEG-A1 injection (6.8 ng) or vehicle, placed in hyperoxia on P7 and prepared for analysis on P9. Retinal vessels were visualized by lectin labeling and the AVA (dotted outline) was quantified using image J. AVA in the PEG-A1 treated retinas was not significantly (n.s.) different from the vehicle controls. Scale bar = 100 µm.

(D, E) PEG-A1 treatment increased A1 protein expression in the P13 OIR retinas.

(F, G) PEG-A1 treatment increased the number of ameboid, Iba1/lectin double-positive microglia/macrophages in the OIR retinas at P9. Scale bar = 100 µm.

(H, I) PEG-A1 treatment reduced VEGF protein levels in OIR retinas at P14 as compared to vehicle treatment.

(J, K) PEG-A1 treatment reduced iNOS protein levels in OIR retinas at P13 as compared to vehicle treatment.

S3

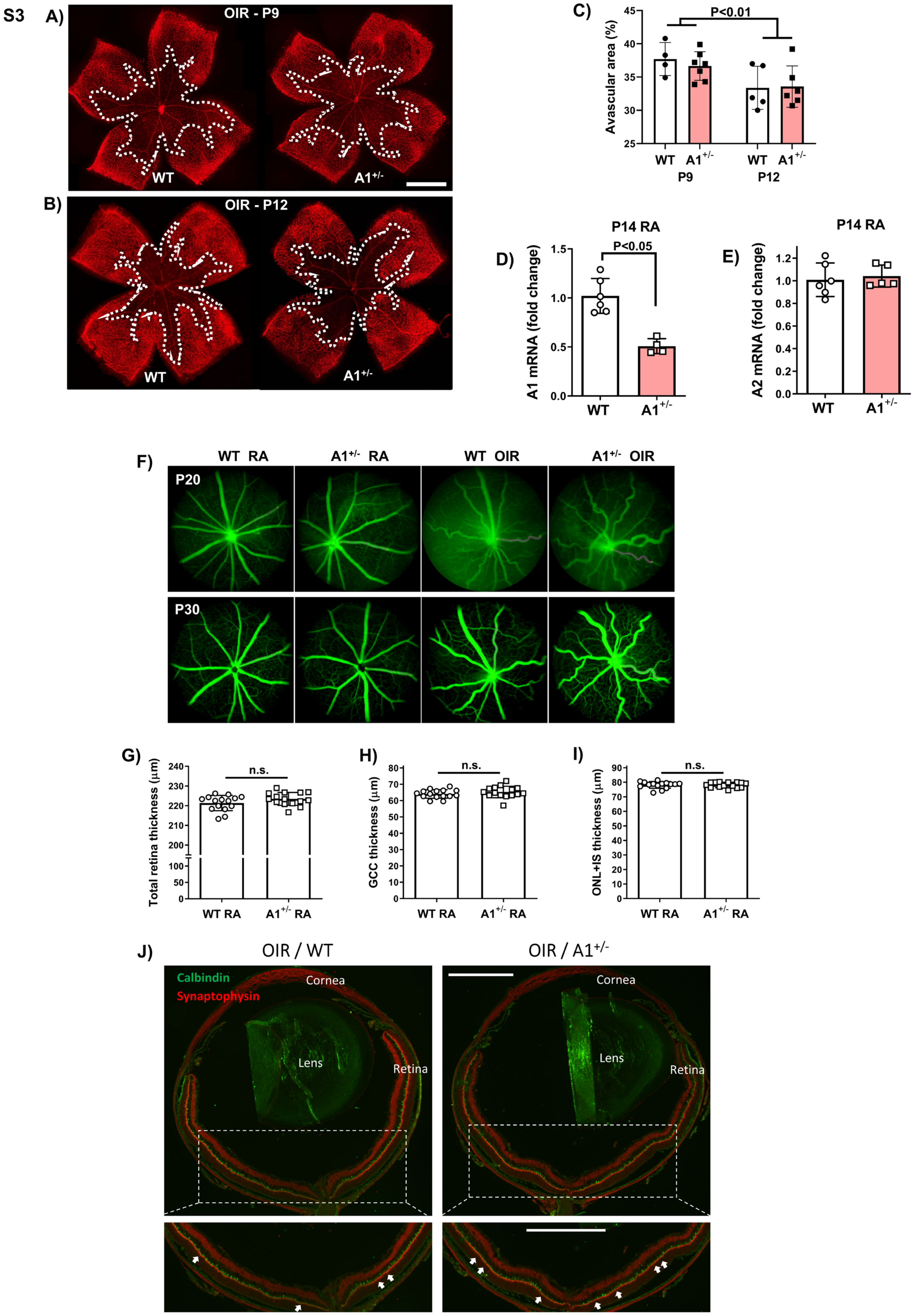
A)
OIR - P9
WT
A1+/-
B)
OIR - P12
WT
A1+/-
C)
P<0.01
Avascular area (%)
45
40
35
30
25
WT
A1+/-
WT
A1+/-
P9
P12
D)
P14 RA
P<0.05
A1 mRNA (fold change)
1.5
1.0
0.5
0.0
WT
A1+/-
E)
P14 RA
A2 mRNA (fold change)
1.2
1.0
0.8
0.6
0.4
0.2
0.0
WT
A1+/-
F)
WT RA
A1+/- RA
WT OIR
A1+/- OIR
P20
P30
G)
n.s.
Total retina thickness (μm)
240
230
220
210
200
100
50
0
WT RA
A1+/- RA
H)
n.s.
GCC thickness (μm)
80
70
60
50
40
30
20
10
0
WT RA
A1+/- RA
I)
n.s.
ONL+IS thickness (μm)
90
80
70
60
50
40
30
20
10
0
WT RA
A1+/- RA
J)
OIR / WT
OIR / A1+/-
Calbindin
Synaptophysin
Cornea
Lens
Retina
Cornea
Lens
Retina

Fig. S3: Supporting data from WT and A1$^{+/-}$ mice.

(A-C) WT and A1$^{+/-}$ littermate pups were placed in hyperoxia on P7 and prepared for analysis on P9 or P12. Retinal vessels were visualized by lectin labeling and the avascular area (AVA) (dotted outline) was quantified using image J. AVA of A1$^{+/-}$ retinas was not significantly (n.s.) different from WT control at P9 or P12. Scale bar = 100 μm.

(D, E) As expected, A1$^{+/-}$ retinas at P14 under RA showed a 50% decrease in A1 expression compared to WT retinas with no change in A2 mRNA levels.

(F) Representative fluorescein angiography images of P20 and P30 mice pertaining to fig. 1F and showing increased tortuosity in the A1$^{+/-}$ group.

(G-I) SD-OCT analysis of 12-weeks old WT and A1$^{+/-}$ mice RA retinas showed no difference in thickness of total retina, ganglion cell complex (GCC), and outer nuclear layer plus inner segments (ONL+IS).

(J) Low magnification images of eye globe cross-sections showing loss of horizontal cells in WT OIR retinas which was aggravated in A1$^{+/-}$ OIR retinas. Horizontal cells were immunolabeled with calbindin (green) and retinas were counterstained with the synaptic marker, synaptophysin (red). Arrows indicate areas of horizontal cell loss. Scale bar: 500 μm.

S4

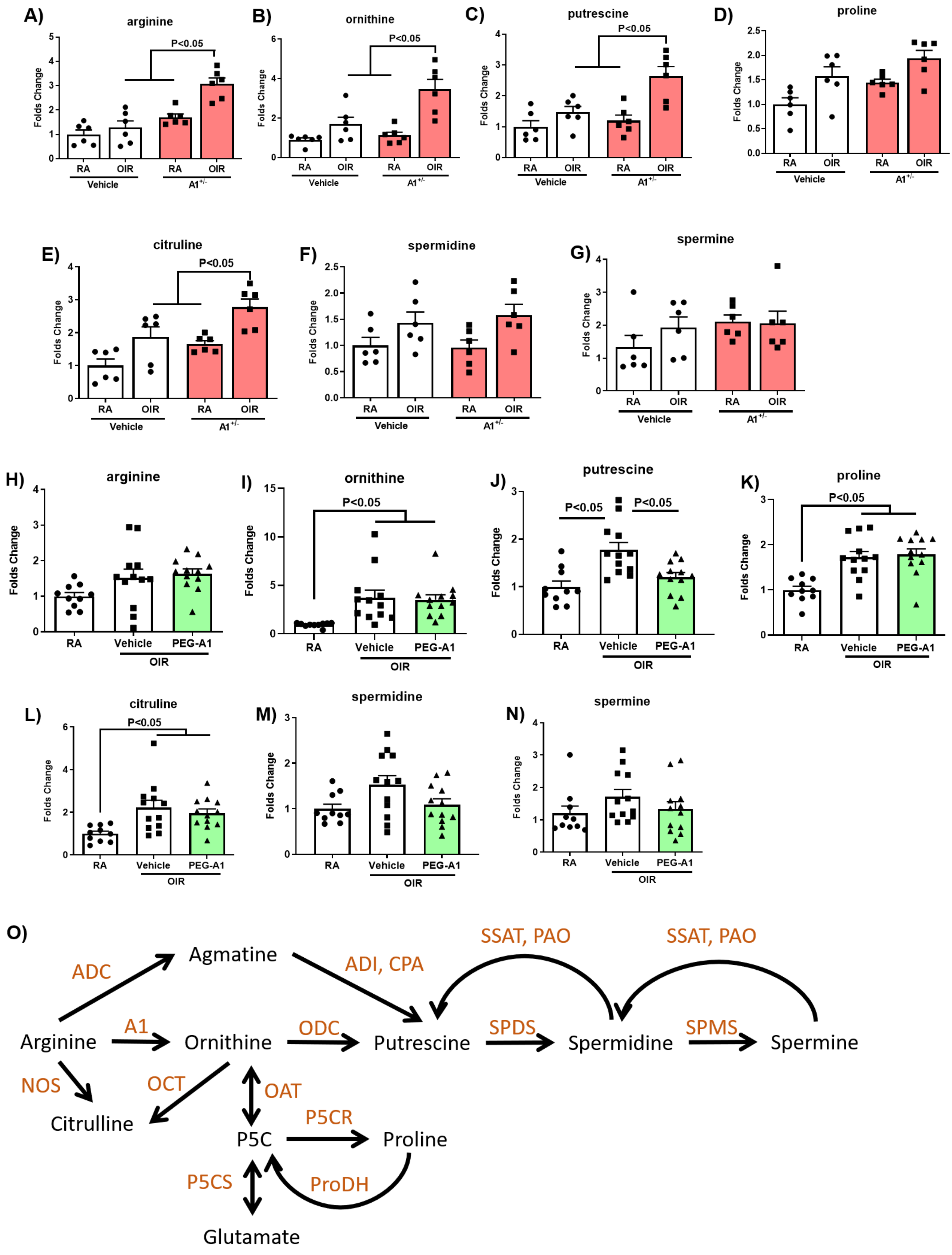
A)
arginine
P<0.05
Folds Change
RA
OIR
RA
OIR
Vehicle
A1+/-
B)
ornithine
P<0.05
Folds Change
RA
OIR
RA
OIR
Vehicle
A1+/-
C)
putrescine
P<0.05
Folds Change
RA
OIR
RA
OIR
Vehicle
A1+/-
D)
proline
Folds Change
RA
OIR
RA
OIR
Vehicle
A1+/-
E)
citruline
P<0.05
Folds Change
RA
OIR
RA
OIR
Vehicle
A1+/-
F)
spermidine
Folds Change
RA
OIR
RA
OIR
Vehicle
A1+/-
G)
spermine
Folds Change
RA
OIR
RA
OIR
Vehicle
A1+/-
H)
arginine
Folds Change
RA
Vehicle
PEG-A1
OIR
I)
ornithine
P<0.05
Folds Change
RA
Vehicle
PEG-A1
OIR
J)
putrescine
P<0.05
P<0.05
Folds Change
RA
Vehicle
PEG-A1
OIR
K)
proline
P<0.05
Folds Change
RA
Vehicle
PEG-A1
OIR
L)
citruline
P<0.05
Folds Change
RA
Vehicle
PEG-A1
OIR
M)
spermidine
Folds Change
RA
Vehicle
PEG-A1
OIR
N)
spermine
Folds Change
RA
Vehicle
PEG-A1
OIR
O)
Agmatine
ADC
ADI, CPA
SSAT, PAO
SSAT, PAO
Arginine
A1
Ornithine
ODC
Putrescine
SPDS
Spermidine
SPMS
Spermine
NOS
OCT
OAT
Citrulline
P5CR
P5C
Proline
P5CS
ProDH
Glutamate

Fig. S4: LC-MRM MS analysis of retinas from A1$^{+/-}$ mice and after PEG-A1 treatment.

(A-G) Liquid chromatography mass spectrometry analysis on OIR retinas showed increased arginine, ornithine, putrescine, and citrulline levels in the A1 KO OIR retinas.

(H-N) Analysis of PEG-A1 treated retinas showed no change in arginine, ornithine, or citrulline levels while putrescine was decreased.

(O) A schematic diagram depicting the arginase polyamine pathway. A1: arginase, ADC: arginine decarboxylase, ODC: ornithine decarboxylase, SPDS: spermidine synthase, SPMS: spermine synthase, NOS: nitric oxide synthase, OCT: Ornithine transcarbamylase, ADI: agmatine deiminase, CPA: N-carbamoylputrescine amidohydrolase, SSAT: Spermidine/spermine-N(1)-acetyltransferase, PAO: polyamine oxidase, OAT: Ornithine δ-aminotransferase, P5CS: Pyrroline-5-carboxylate synthase, P5CR: Pyrroline-5-carboxylate reductase, ProDH: proline dehydrogenase.

S5

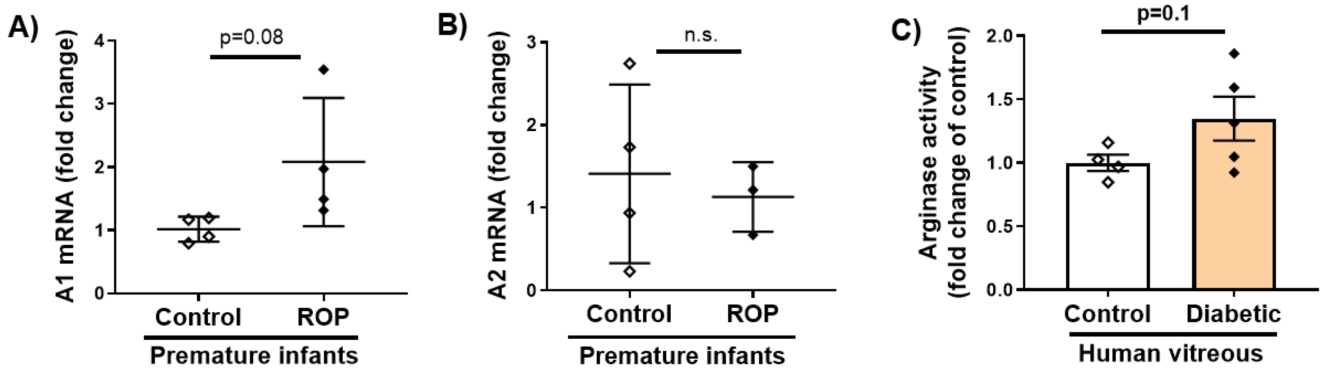

Fig. S5: A1 and A2 expression in human RNV conditions.

(A, B) A1 but not A2 mRNA was increased in blood samples from ROP infants although this was not statistically significant.

(C) Arginase activity the vitreous collected from diabetic retinopathy (DR) patients showed an increasing trend although the alteration was not statistically significant.

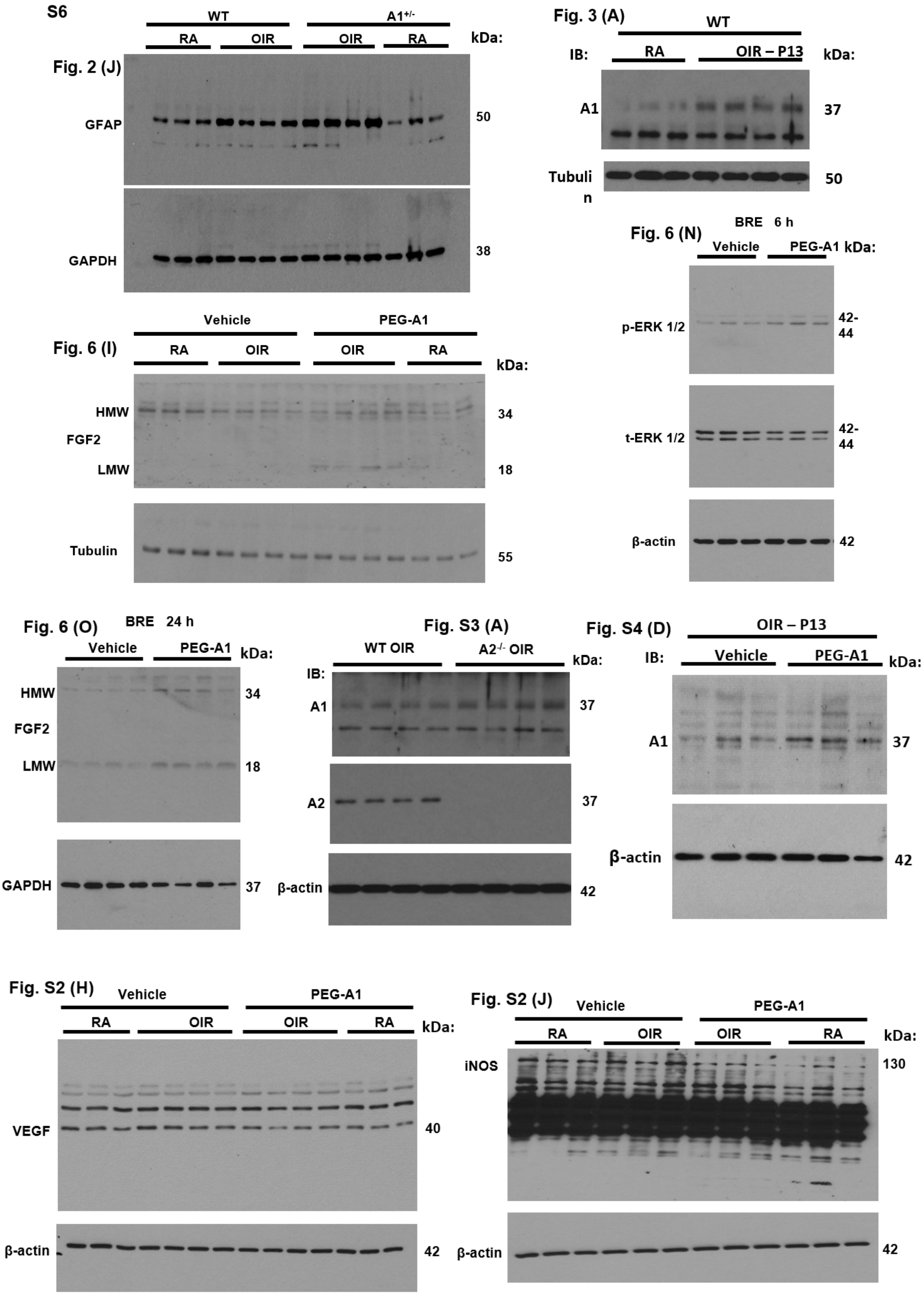

Fig. S6: Original un-cropped pictures of the Western blots presented in the article.

Graphical abstract

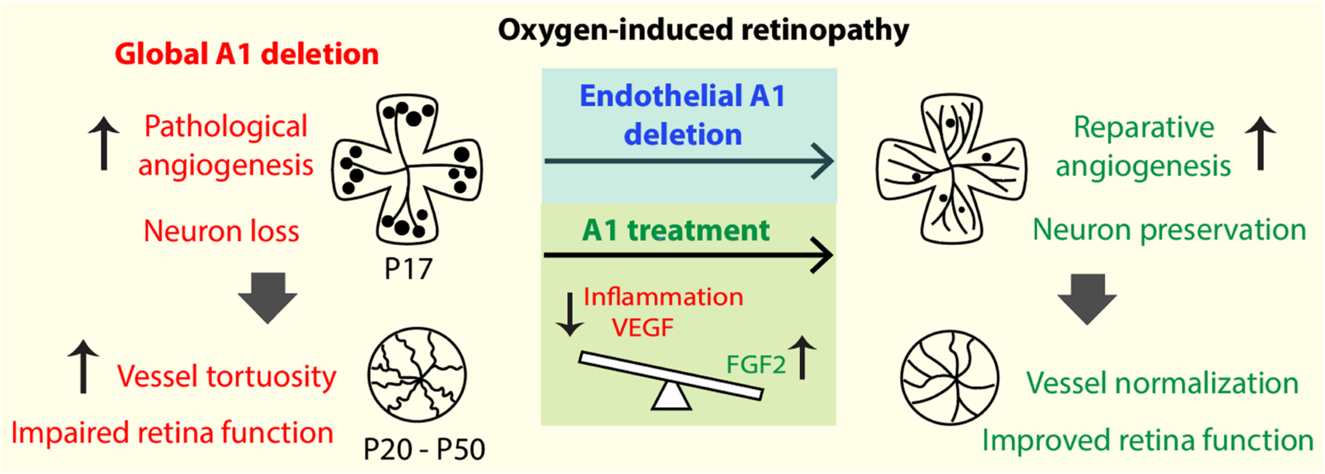

References:

1. Fouda AY, Xu Z, Shosha E, Lemtalsi T, Chen J, Toque HA, et al. Arginase 1 promotes retinal neurovascular protection from ischemia through suppression of macrophage inflammatory responses. Cell death & disease. 2018;9(10):1001.

2. Connor KM, Krah NM, Dennison RJ, Aderman CM, Chen J, Guerin KI, et al. Quantification of oxygen-induced retinopathy in the mouse: a model of vessel loss, vessel regrowth and pathological angiogenesis. Nature protocols. 2009;4(11):1565-73.

3. Patel C, Narayanan SP, Zhang W, Xu Z, Sukumari-Ramesh S, Dhandapani KM, et al. Activation of the endothelin system mediates pathological angiogenesis during ischemic retinopathy. Am J Pathol. 2014;184(11):3040-51.

4. Mezu-Ndubuisi OJ. In Vivo Angiography Quantifies Oxygen-Induced Retinopathy Vascular Recovery. Optom Vis Sci. 2016;93(10):1268-79.

5. Narayanan SP, Xu Z, Putluri N, Sreekumar A, Lemtalsi T, Caldwell RW, et al. Arginase 2 deficiency reduces hyperoxia-mediated retinal neurodegeneration through the regulation of polyamine metabolism. Cell death & disease. 2014;5(2):e1075-e.

6. Shosha E, Xu Z, Yokota H, Saul A, Rojas M, Caldwell RW, et al. Arginase 2 promotes neurovascular degeneration during ischemia/reperfusion injury. Cell death & disease. 2016;7(11):e2483.

7. Narayanan SP, Suwanpradid J, Saul A, Xu Z, Still A, Caldwell RW, et al. Arginase 2 deletion reduces neuro-glial injury and improves retinal function in a model of retinopathy of prematurity. PloS one. 2011;6(7):e22460.

8. Prusky GT, Alam NM, Beekman S, Douglas RM. Rapid quantification of adult and developing mouse spatial vision using a virtual optomotor system. Investigative ophthalmology & visual science. 2004;45(12):4611-6.

9. Douglas RM, Alam NM, Silver BD, McGill TJ, Tschetter WW, Prusky GT. Independent visual threshold measurements in the two eyes of freely moving rats and mice using a virtual-reality optokinetic system. Visual neuroscience. 2005;22(5):677-84.

10. Behzadian MA, Wang XL, Jiang B, Caldwell RB. Angiostatic role of astrocytes: suppression of vascular endothelial cell growth by TGF-beta and other inhibitory factor(s). Glia. 1995;15(4):480-90.

11. Shao Z, Friedlander M, Hurst CG, Cui Z, Pei DT, Evans LP, et al. Choroid sprouting assay: an ex vivo model of microvascular angiogenesis. PloS one. 2013;8(7):e69552.

12. Tomita Y, Shao Z, Cakir B, Kotoda Y, Fu Z, Smith LEH. An Ex Vivo Choroid Sprouting Assay of Ocular Microvascular Angiogenesis. Journal of visualized experiments : JoVE. 2020(162).
